# Improvements in skilled walking associated with kinematic adaptations in people with spinal cord injury

**DOI:** 10.1186/s12984-019-0575-z

**Published:** 2019-08-28

**Authors:** Raza N. Malik, Gevorg Eginyan, Andrea K. Lynn, Tania Lam

**Affiliations:** 1 School of Kinesiology, University of British Columbia, 210-6081 University Boulevard, Vancouver, BC V6T 1Z1 Canada; 2 International Collaboration on Repair Discoveries, University of British Columbia, 818 West 10th Avenue, Vancouver, BC V5Z 1M9 Canada

**Keywords:** Inter-joint coordination, Skilled walking, Spinal cord injury, Rehabilitation, Locomotor training, Kinematics, Community ambulation, Sensorimotor integration

## Abstract

**Introduction:**

Individuals with motor-incomplete SCI (m-iSCI) remain limited community ambulators, partly because they have difficulty with the skilled walking requirements of everyday life that require adaptations in inter-joint coordination and range of motion of the lower limbs. Following locomotor training, individuals with SCI show improvements in skilled walking and walking speed, however there is limited understanding of how adaptations in lower limb kinematics following training contribute to improvements in walking.

**Objective:**

To determine the relationship between changes in lower limb kinematics (range of motion and inter-joint coordination) and improvements in walking function (walking speed and skilled walking) following locomotor training.

**Methods:**

Lower limb kinematics were recorded from 8 individuals with chronic m-iSCI during treadmill walking before and after a 3-month locomotor training program. Data were also collected from 5 able-bodied individuals to provide normative values. In individuals with SCI, muscle strength was used to define the stronger and weaker limb. Motion analysis was used to determine, hip, knee and ankle angles. Joint angle-angle plots (cyclograms) were used to quantify inter-joint coordination. Shape differences between pre-and post-training cyclograms were used to assess the changes in coordination and their relation to improvements in walking function. Walking function was assessed using the 10MWT for walking speed and the SCI-FAP for skilled walking. Comparing pre- and post-training cyclograms to the able-bodied pattern was used to understand the extent to which changes in coordination involved the recovery of normative motor patterns.

**Results:**

Following training, improvements in skilled walking were significantly related to changes in hip-ankle coordination (*ρ* = − .833, *p* = 0.010) and knee range of motion (*ρ* = .833, *p* = 0.010) of the weaker limb. Inter-joint coordination tended to revert towards normative patterns, but not completely. No relationships were observed with walking speed.

**Conclusion:**

Larger changes in hip-ankle coordination and a decrease in knee range of motion in the weaker limb during treadmill walking were related to improvements in skilled walking following locomotor training in individuals with SCI. The changes in coordination seem to reflect some restoration of normative patterns and the adoption of compensatory strategies, depending on the participant.

## Introduction

Rehabilitation and exercise training programs are important for individuals with spinal cord injury (SCI), not only to promote mobility, but also for minimizing the effects of secondary complications and improving quality of life [1–4]. Following motor-incomplete SCI (m-iSCI), individuals have reduced muscle strength [5, 6], inadequate range of motion [7] and impaired inter-joint coordination [8, 9], all factors that likely contribute to the increased falls-risk [10] and limited community ambulation in this population [11, 12]. Gait rehabilitation strategies for people with m-iSCI have been built upon principles of providing appropriate (walking-related) afferent feedback to central locomotor circuits (‘pattern generators’) within the spinal cord [13–16]. Systematic reviews of repetitive practice of walking, whether over-ground or on a treadmill, and assisted by robotics or a therapist, have revealed modest improvements in lab-based measures of walking speed and endurance [17–20] that do not necessarily translate to better community ambulation [11, 12].

Multi-joint movements, whether reaching for a cup or stepping over an obstacle, are essential components of everyday life. These skilled movements require concurrent rotations about several joints to guide the end-point along a desired trajectory towards or over a target. During walking, accurate control of foot trajectory during the swing phase is necessary for ensuring foot-floor or foot-object clearance to avoid tripping and falling. The precise control of foot trajectory requires simultaneous movement and coordination of multiple joints of the lower limb [21–26]. Following a neurological injury an individual may deploy stiff-gait strategies to minimize the inter-joint coordination required to successfully achieve adequate foot-floor clearance during over ground walking [27–29]. However, reliance on such strategies will limit locomotor adaptability. An obvious example is stepping over obstacles, which requires greater flexion at the hip and knee compared to unobstructed walking [21]. To step over an obstacle, the nervous system reorganizes its strategy [30, 31] and relies more on intersegmental dynamics to control swing limb trajectory [21]. Lessons from reaching studies [32] indicate that as the need to control for inter-segmental dynamics increases (as in obstacle crossing), the greater the emphasis on inter-joint coordination, compared to that required to produce simpler movement trajectories (as in regular walking over a level surface).

Investigating the association between lower limb kinematics and walking function provides insight into understanding how the nervous system adapts its motor control strategies to improve walking function after a locomotor intervention. Following locomotor training, improvements in hip [33] or ankle [34] range of motion have been reported alongside improvements in walking speed. Indeed, adaptations to enable faster walking speeds are achieved by increasing joint range of motion [35, 36], especially at the hip [9]. Although walking speed can be increased with training, this is not necessarily accompanied by improvements in motor control, such as those indicated by measures of inter-joint coordination. Nooijen et al. [37] quantified inter-joint coordination by examining the onset of knee extension with respect to the onset of hip flexion, and reported no changes in this parameter following 12 weeks of locomotor training [37]. Inter-joint coordination has also been quantified by examining the spatial trajectory of the hip and knee joints simultaneously (angle-angle plots) and comparing it to normative, able-bodied coordination patterns [8, 9]. Improvements in walking speed were shown in individuals with iSCI following inpatient rehabilitation, but these improvements were not associated with the re-establishment of normative hip-knee coordination patterns [9]. Thus, the findings to-date suggest that, at least for regular walking speed measured on a flat surface, changes in lower limb coordination may not be critical for recovery of overground walking speed.

As reviewed above, the regulation of multi-segmental control by the locomotor system is important for skilled walking tasks, such as obstacle crossing. In this study, we sought to contrast the relationship between changes in lower limb kinematics (joint range of motion and inter-joint coordination) and walking function (skilled walking and walking speed) following locomotor training. Skilled walking was assessed using the Spinal Cord Injury Functional Ambulation Profile (SCI-FAP) and walking speed was assessed using the 10-m walk test (10MWT). We hypothesized that larger changes in lower limb inter-joint coordination control following training would be related to improvements in skilled walking, but not walking speed; and that greater joint range of motion following training would be related to improvements in both walking skill and speed.

## Methods

### Participants

Kinematic data from individuals with m-iSCI who participated in a previously published pilot randomized control trial [38] were included. All participants with SCI had chronic injuries (> 12 months) at or above T11 spinal level and were able to walk on a treadmill with body-weight support (BWS) but without manual assistance. All participants provided written consent and all procedures were approved by the University of British Columbia Clinical Research Ethics Board.

We were only able to include data from 8 of the original 13 participants who completed the pilot RCT. Data from the other 5 individuals from the previous study were excluded because of missing kinematic data, due to either technical difficulties or missed recording sessions. We also recruited five able-bodied (AB) controls to provide normative kinematic data.

### Protocol

For individuals with SCI, kinematic data from the lower limbs were recorded during treadmill walking before and after a 3-month Lokomat-based training program. Details of the training program are described elsewhere [38], but briefly, participants underwent 45 min of Lokomat-based training three times per week for three-months. Sagittal-plane kinematic data of the hip, knee, and ankle joints were recorded before and after training using the Optotrak motion capture system (Northern Digital Inc., Waterloo, ON, Canada) while participants walked on a treadmill with a safety harness. The amount of BWS provided (Table 1) was the minimum amount of support required while ensuring proper stance phase kinematics (i.e. hip and knee extension) when walking with the Lokomat. Treadmill speed and the amount of body-weight support were matched between pre- and post-training recording sessions (test parameters are listed in Table 1). Infrared-emitting diodes were placed bilaterally on the greater trochanter, lateral epicondyle of the knee, lateral malleolus, and hallux. All data were recorded at 100 Hz.

**Table 1:** SCI participant characteristics, pre-training measurements, and treadmill walking test parameters

Participant Characteristics							Pre-Training Measurements						Test Parameters		
Participant Code	Sex	Age (years)	AIS	Injury Level	Etiology	Chronicity (years)	Strong Step Length (cm)	Weak Step Length (cm)	Strong LEMS	Weak LEMS	SCI-FAP	10 MWT (m/s)	Treadmill Speed (m/s)	BW unloaded (% of body mass)	# of steps (pre, post)
SCI01	M	31	D	T4	Trauma	6	35.06	36.68	14	1	601	0.09	0.06	12	12, 12
SCI02	M	57	D	C4	Trauma	2	60.53	56.65	24	15	365	0.15	0.29	8	23, 23
SCI03	M	34	C	C4–5	Trauma	5	60.50	58.80	9	9	176	0.24	0.32	0	15, 16
SCI04	M	38	D	C1/2	Trauma	4	67.05	68.71	23	14	88	0.59	0.13	0	22, 37
SCI05	F	53	D	T3	Hemorrhage	2	54.02	54.90	23	10	83	0.34	0.23	7	19, 31
SCI06	M	63	D	C5	Trauma	2	53.54	55.50	23	17	58	0.33	0.39	0	27, 30
SCI07	F	27	D	T10	AVM	15	62.26	63.40	14	11	38	0.84	0.71	0	55, 39
SCI08	F	26	D	C4/5	Trauma	3	78.51	71.58	24	17	8	1.03	1.04	7	63, 63
–	5M;3F	36	7D;1C	–	–	3.5	60.51	57.73	23	12.5	85.5	0.34	0.31	3.5	22.5, 30.5

Strong and weak is determined by LEMS score

Numerical values in the last row represent the median

*AIS* American spinal cord injury association impairment scale, *LEMS* Lower extremity motor score, *SCI-FAP* Spinal cord injury functional ambulation profile, *10MWT* 10-meter walk test, *BW* Body weight

Normative data from the AB control participants were collected during a single session of treadmill walking. AB participants walked at different combinations of speed and body-weight support that matched the treadmill walking parameters of each SCI participant. Lower limb kinematics were collected as described above.

For individuals with SCI, the change in walking function was defined by skilled walking capacity and walking speed, which were measured pre- and post-training intervention. Walking speed was measured using the 10-m walk test (*10MWT*) [39]. Participants were instructed to walk 10 m at their maximum speed. Higher values indicate faster walking speeds. Skilled walking capacity was measured using the Spinal Cord Injury-Functional Ambulation Profile (*SCI-FAP*). The SCI-FAP is a series of seven timed walking tasks which include negotiating obstacles, curbs and stairs among other functionally relevant walking tasks [40]. Lower SCI-FAP scores indicate better skilled walking capacity. Both measures have been shown to be valid and reliable methods of examining walking function in individuals with SCI [40–42].

The Lower Extremity Motor Score (LEMS) was also used to determine lower limb muscle strength in participants with SCI [43]. This assessment was conducted by the same physiotherapist for all SCI participants.

### Data analysis

Kinematic data were analyzed offline using MATLAB (Mathworks Inc., Natick, MA, USA). Data were filtered using a low-pass 4th order Butterworth filter with a cut off frequency of 6 Hz. Individual steps were defined from foot contact to foot contact, determined by the anterior-posterior displacement of the toe marker. For individuals with SCI, the stronger limb was defined as the limb with the higher LEMS, and the weaker limb was defined as the limb with the lower LEMS at pre-training. In SCI03, who had the same LEMS on the left and right, the weaker limb was defined as the limb with the shorter step-length [9, 34, 36, 44].

#### Walking Function

The percent change in pre- and post-training SCI-FAP and 10MWT measurements were used to quantify the change in skilled walking and walking speed, respectively. For the SCI-FAP, negative values indicate an improvement in skilled walking function. For the 10MWT, positive values indicate an improvement in walking speed.

#### Range of motion (ROM)

**Fig. 1:**
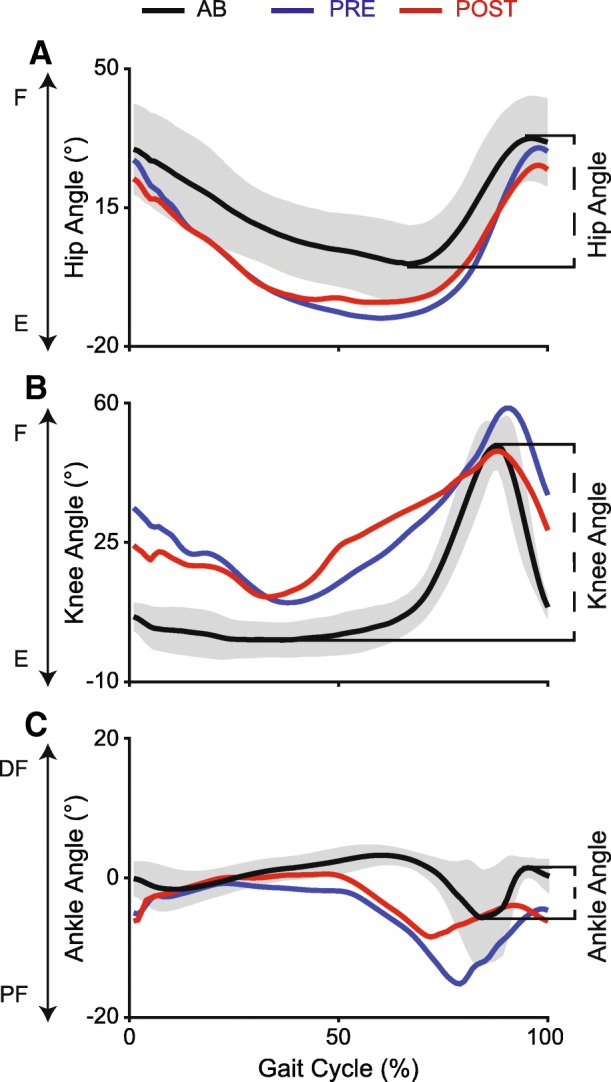
Hip (**a**), knee (**b**), and ankle angles (**c**) of the weaker limb are shown pre- and post-training for SCI04. Joint angles averaged across all AB participants at the matched speed and BWS are also shown. The grey shaded area around the AB average represents the standard deviation. AB, able-bodied; SCI, spinal cord injury; BWS, Body-weight support; F, flexion; E, extension; DF, dorsi-flexion; PF, plantar-flexion

Sagittal plane hip, knee, and ankle joint angles were derived from the marker positions using custom-written MATLAB code. For individuals with SCI, joint angles were averaged across all steps (Table 1) for each limb to determine the average joint excursions for the stronger and weaker limb during walking. For able-bodied participants, joint angles were averaged across all steps and between left and right to obtain normative joint excursion data during walking. Hip ROM (*ROM*_*H*_) and knee ROM (*ROM*_*K*_) were calculated by subtracting the average minimum angle during the gait cycle from the average maximum achieved during swing (Fig. 1a and b). Ankle ROM (*ROM*_*A*_) was calculated by subtracting the average ankle angle at toe off from the average ankle angle at mid-swing (Fig. 1c). To determine the change in ROM with locomotor training, pre-training ROM values were subtracted from post-training values. Larger values indicate greater change in ROM at post-training.

#### Inter-joint coordination

Inter-joint coordination, examining hip-knee (*HK*), hip-ankle (*HA*) and knee-ankle (*KA*) coordination, was determined by comparing pre- and post-training angle-angle plots. Following the method described by Awai and Curt [8], joint angles were first translated and scaled. Joint angles were scaled by subtracting the minimum value, effectively making the lowest value 0. Data were then scaled by their maximum value and translated to their centroid by subtracting the mean. This process centers the cyclograms around the origin of the axes and removes any differences due to joint range of motion, allowing for comparison of the shape difference in the coordination patterns [8]. The shape difference between pre- and post-training cyclograms [8] was calculated by the square root of the sum of squared differences (SSD) between cyclograms (*SSD*_*pre-post*_). Thus, if pre- and post-training cyclograms are identical, the SSD_pre-post_ score will be 0. Larger values indicate a greater change in coordination patterns at post-training.

To evaluate differences in inter-joint coordination with respect to a ‘normative’ pattern, the SSD was also calculated between the reference cyclograms from the AB group and the cyclograms of SCI participants at pre-training (*SSDnorm*_*pre*_) and post-training (*SSDnorm*_*post*_). The reference cyclogram from the AB group was drawn from the appropriate combination of treadmill speed and BWS used by each SCI participant. Here, SSD scores of 0 would indicate no difference between the SCI and AB cyclograms.

### Statistical analysis

Given the small sample size of this data set we employed non-parametric analyses. Descriptive statistics were used to describe the characteristics of each group. Spearman’s rho (ρ) was used to determine the relationship between changes in lower limb kinematic parameters and walking function. We calculated ρ between SSDHK_pre-post_, SSDHA_pre-post_ and SSDKA_pre-post_ against percent change in SCI-FAP and 10MWT. The same analysis was performed for ROM_H_, ROM_K_ and ROM_A_ against percent change in SCI-FAP and 10MWT. Normalized single participant data (*SSDnorm*_*pre*_, *SSDnorm*_*post*_*)* were presented descriptively to evaluate whether inter-limb coordination was reinstated to the AB pattern following training. To account for multiple correlations of the SCI-FAP and 10MWT with each set of kinematic variables, we used the Bonferroni adjusted alpha value of 0.016 (0.05/3) to evaluate statistical significance. All statistical analyses were conducted using SPSS v.20 statistics (IBM, Armonk, NY).

## Results

### Participant characteristics

AB and SCI participant characteristics and treadmill test parameters are reported in Table 1. For AB individuals, the median age was 26 years (range: 22–29). For individuals with SCI, the median age was 36 years (range: 26–63) and the median time post-injury was 3.5 years (range: 2–15). There was one individual with AIS C and 7 individuals with AIS D. The median percentage of body weight unloaded during walking was 3% (range: 0–12) and the median treadmill walking speed used was 0.31 m/s (range: 0.06–1.04). Pre- and post-training changes in the SCI-FAP and 10MWT are listed in Table 2 for each participant.

**Table 2:** SCI participants' functional gait measures

Participant Code	Pre-Training SCI-FAP Score	Post-Training SCI-FAP Score	SCI-FAP % Change	Pre-Training 10 MWT (m/s)	Post-Training 10 MWT (m/s)	10 MWT % Change
SCI01	600.51	403.15	−32.86	0.09	0.13	44.44
SCI02	365.21	270.35	− 25.97	0.15	0.22	46.67
SCI03	175.58	193.17	10.01	0.24	0.32	33.33
SCI04	87.60	56.35	−35.67	0.59	0.89	50.85
SCI05	82.68	65.53	−20.74	0.34	0.43	26.47
SCI06	58.36	54.47	−6.66	0.33	0.66	100.00
SCI07	37.70	36.36	−3.56	0.84	0.86	2.38
SCI08	8.44	7.97	−5.52	1.03	1.11	7.77
–	85.14	60.94	−13.70	0.34	0.55	38.89

Last row represents the median

*SCI-FAP* Spinal cord injury functional ambulation profile, *10MWT* 10-meter walk test

### Changes in inter-joint coordination following training

**Fig. 2:**
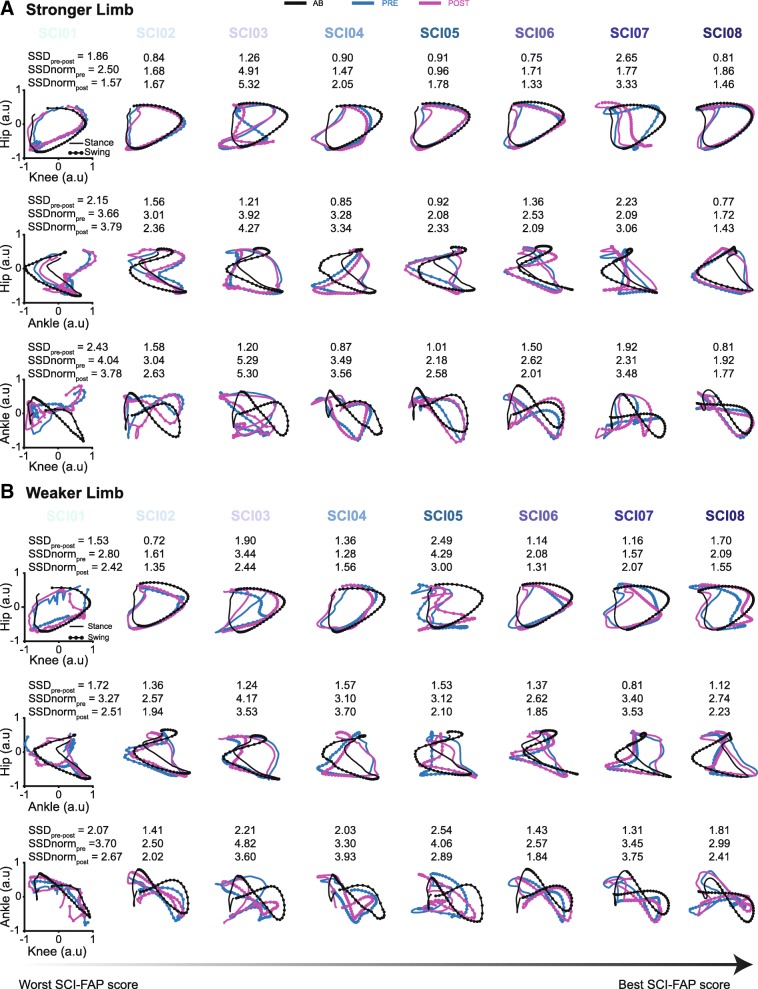
Joint angle-angle plots (cyclograms) depicting hip-knee (HK; top plots), hip-ankle (HA; middle plots), and knee-ankle (KA; bottom plots) inter-joint coordination patterns for each SCI participant pre- and post-training for both the stronger (**a**) and weaker limb (**b**). Each set of plots includes the average AB reference data (black lines) matched for the speed and BWS used by the SCI participant. Plots are ordered from left to right by worst to best SCI-FAP score among the SCI participants. Angle-angle plots were used to quantify the change in inter-joint coordination patterns by calculating the square root of sum of squared differences (SSD) between cyclograms before and after training for each participant (SSD_pre-post_). We also compared each SCI participants’ pre- and post-training cyclograms to the AB cyclogram (SSDnorm_pre_ and SSDnorm_post_) to determine the extent to which SCI individuals re-established the AB coordination pattern following training. These scores are presented above each set of cyclograms. Bigger SSD scores represent a greater difference between the cyclograms being compared. Positive values indicate flexion/dorsiflexion for all joints. AB, able-bodied; SCI, spinal cord injury; a.u, arbitrary units; SCI-FAP, Spinal Cord Injury Functional Ambulation Profile

In Fig. 2, HK (top), HA (middle) and KA (bottom) cyclograms from each participant at pre-and post-training are shown for both the stronger (Fig. 2a) and weaker limb (Fig. 2b) and are plotted in rank from left to right by worst to best pre-training SCI-FAP score. The associated cyclograms from the averaged AB data are also plotted to provide reference normative data. Overall, each participant showed individualized changes in their cyclograms at post-training (blue vs. red traces, Fig. 2). It also appeared that individuals who had the greatest impairment in skilled walking function at pre-training tended to have the most apparent changes in their cyclograms at post-training (SSD_pre-post_ values indicated in Fig. 2).

As mentioned below, the change in HA coordination of the weaker limb from pre- to post-training was most associated with improvements in the SCI-FAP score. In Fig. 2b we observed some interesting changes in HA coordination of the weaker limb across participants. For SCI01, we noted that following training the hip and ankle form a cyclogram, which was not present prior to training. In SCI02, SCI04 and SCI06, we observed the formation of a “loop” at the transition from swing to stance, which was not as prominent prior to training. In SCI03 and SCI05, we observed a change in the shape of the cyclogram after training especially during the swing phase. Moreover, SCI05’s cyclogram after training showed fewer straight lines and more curved and rounded lines, indicating that after training the hip and ankle were moving simultaneously, rather than sequentially. SCI07 and SCI08, who were the higher-functioning participants among our cohort, tended to have minimal changes in the overall shape of the cyclogram between pre- and post-training.

**Fig. 3:**
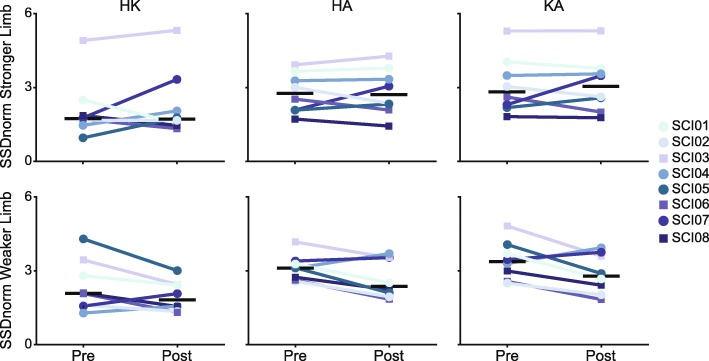
SSD scores comparing pre- and post-training cyclograms to the AB cyclogram (SSDnorm_pre_ and SSDnorm_post_) for the hip-knee (HK, left plots), hip-ankle (HA, middle plots), and knee-ankle (KA, right plots) coordination patterns are shown for each SCI participant for both the stronger (top plots) and weaker limb (bottom plots). Black horizontal lines represent the median. SSDnorm scores of 0 would indicate no difference between the SCI and AB cyclograms. SSD, square root of sum of squared differences; AB, able-bodied; SCI, spinal cord injury

A comparison of pre- and post-training SSD scores normalized to the AB reference values show that SSDnorm scores in the weaker limb approached zero following training, indicating a reversion towards normative inter-joint coordination patterns in the weaker limb (Fig. 3 bottom panels). In the stronger limb, SSDnorm scores did not seem to approach zero, suggesting no appreciable reinstatement of normative inter-joint coordination patterns (Fig. 3 top panels). Taking a closer look at HA coordination, as it was significantly associated with improvements in skilled walking, 6 of the 8 participants showed a decrease in the SSDnorm score following training. Most notably, HA cyclograms following training in SCI01 and SCI02 appeared to become more similar to the AB cyclograms, although differences still remained (Fig. 2b middle panel). Improvements in skilled walking were also seen in participants who did not appear to show similar cyclograms to the AB cyclograms following training, most notably SCI04 (Fig. 2b middle panel).

### Relationship between changes in walking function and kinematic parameters

**Fig. 4:**
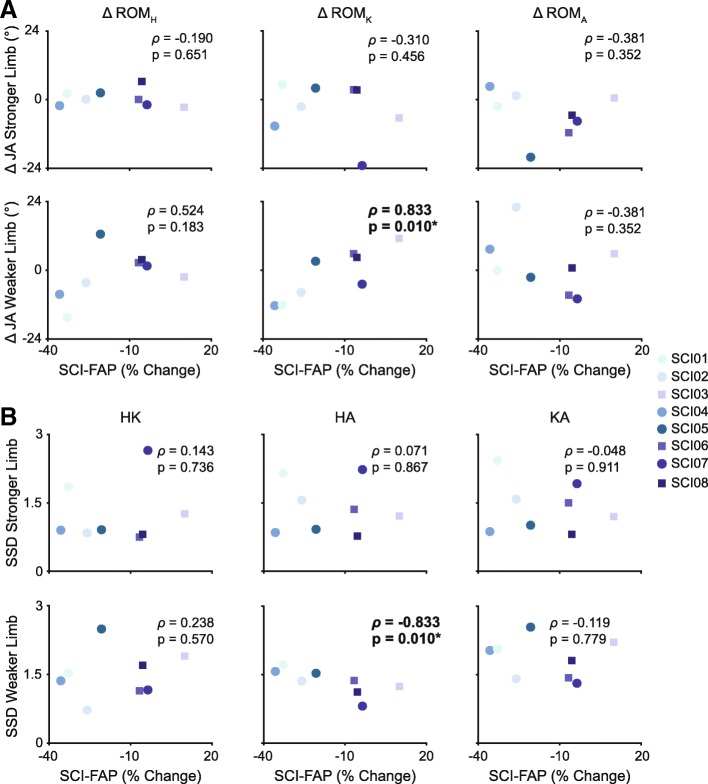
Relationship between changes in kinematics and skilled walking. **a** Changes in range of motion at the hip (ROM_H_, left plots), knee (ROM_K_, middle plots), and ankle (ROM_A_, right plots) are plotted against the changes in skilled walking (SCI-FAP) for both the stronger and weaker limb. **b** SSD scores comparing pre-and post-training cyclograms (SSD_pre-post)_ for the hip-knee (HK, left plots), hip-ankle (HA, middle plots) and knee-ankle (KA, right plots) inter-joint coordination patterns are plotted against the changes in skilled walking (SCI-FAP) for both the stronger and weaker limb. SCI-FAP, Spinal Cord Injury Functional Ambulation Profile; 10MWT; JA, joint angle; SSD, square root of sum of squared differences, ρ; Spearman’s correlation coefficient (rho)

The change in SCI-FAP was significantly associated with ROM_K_ on the weaker side (*ρ* = .833, *p* = 0.010), such that improvements in skilled walking were associated with a reduction in knee range of motion (Fig. 4a, bottom-middle panel). The change in SCI-FAP was also significantly correlated with SSDHA_pre-post_ of the weaker limb (*ρ* = −.833, *p* = 0.010) such that improvements in SCI-FAP were associated with larger changes in HA coordination (Fig. 4b, bottom-middle panel). No other significant relationships were observed.

## Discussion

We found that adaptations in hip-ankle inter-joint coordination and knee ROM of the weaker limb following locomotor training may be related to improvements in skilled walking. The changes in inter-joint coordination of the weaker limb seem to involve some re-establishment of normative motor patterns along with the adoption of compensatory inter-joint coordination strategies, depending on the individual. We did not observe any relationships between any kinematic variables with change in walking speed.

### Kinematic adaptations specific to improvements in skilled walking

Coordinating the joints of the lower limb is important for voluntarily adapting our gait pattern to meet the varied demands of the environment, such as obstacles, curbs, or stairs. Here, we found that larger changes in HA coordination and a decrease in knee range of motion of the weaker limb during treadmill walking were associated with improved skilled walking. To achieve optimal foot clearance, the joints of the lower limb need to work together by utilizing active and passive forces [21, 23, 24, 45]. In most participants, HA coordination in the weaker limb appeared to become more similar to the AB pattern after training. These adaptations in HA coordination and ROM_k_ following training may reflect a change in how the joints of the weaker limb work together to achieve adequate foot clearance during skilled walking. Similar adaptations may not be necessary during uninterrupted waking since we did not observe a relationship between inter-joint coordination and 10MWT. Adequate foot-floor clearance is certainly needed to complete the 10MWT, but this could have been achieved by using compensatory, stiff-gait strategies, which reduce the number of joints needed to be coordinated [27, 28]. In contrast, skilled walking tasks such as obstacle crossing, curb negotiation and climbing a flight of stairs likely require an individual to coordinate multiple joints simultaneously to safely and successfully complete these tasks.

During skilled walking tasks the nervous system adapts its basic locomotor pattern to achieve the desired limb trajectory [21, 30, 31]. During unobstructed walking, muscle activity can be accounted for by five basic patterns associated with different times in the gait cycle [31, 46]. In contrast, during a voluntary gait modification, such as stepping over an obstacle, the underlying locomotor pattern is superimposed with a sixth component appearing just before an individual lifts their limb over an obstacle [31]. In comparison, modifications in walking speed do not change the basic locomotor pattern, rather, only a phase shift is observed due to the changes in the onset of swing [46]. The act of voluntarily lifting the limb over an obstacle not only requires a new sequence of muscle activity [31], but also generates larger motion dependent torques [21]. These changes increase the demand on the nervous system to control for inter-joint coordination to accurately regulate foot clearance for safe skilled walking. In this study, we observed that the SCI individuals who demonstrated the greatest change in inter-joint coordination and ROM pre- to post-training also tended to have the greatest improvements in skilled walking. These kinematic adaptations may reflect a change in the ability to integrate sensorimotor information to control movement, in this case, control of skilled walking.

Contrary to our hypothesis, our data showed that improvements in 10MWT following locomotor were not associated with changes in ROM_H_, ROM_K_ or ROM_A_ in either the stronger or weaker limb. In contrast, Awai and Curt [9] showed that the change in hip ROM (of the more affected leg) was a strong predictor of change in walking speed following in-patient SCI rehabilitation, although hip ROM itself had only a moderate-to-weak relationship to walking speed. In individuals with hemiparesis following stroke, it is the inability to produce adequate hip and ankle flexor power, not joint ROM itself, that limits their ability to increase walking speed [47]. It could be that the ability to increase walking speed is dependent on the power generated at each joint rather than ROM at each joint.

### Are improvements in skilled walking due to compensatory strategies or recovery of normative strategies?

Previous research in SCI [9] and stroke [48] have suggested that improvements in walking speed and arm function, respectively, following rehabilitation are due to compensatory strategies rather than the recovery of normative motor control strategies. In this study, HA SSDnorm_post_ scores in 6 of the 8 participants moved towards zero in the weaker limb. This finding suggests that most participants moved towards re-establishing normative HA coordination patterns. However, it is important to note that the SSDnorm_post_ scores for HA coordination in the weaker limb were still elevated (> 2), and it was evident that the cyclograms were not identical to the AB cyclograms. Moreover, there were participants like SCI04 who showed improvements in skilled walking but whose cyclogram did not revert towards the AB reference data. Our interpretation of this finding is that this participant may rely on compensatory strategies to improve their skilled walking. Thus, our findings suggest that improvements in skilled walking could potentially be from a combination of restoring normative motor control strategies and compensatory coordination strategies depending on the individual.

### Stronger vs. weaker limb

We found that the improvements in walking function seen after the locomotor intervention were associated with changes in inter-joint coordination and ROM only in the weaker limb. Results from the literature on whether the kinematics of the stronger or weaker limb is more affected by locomotor training in individuals with iSCI is limited and variable. Following locomotor training, individuals with iSCI have shown improvements in the kinematics of the stronger limb [33, 44], the weaker limb [33, 34] or neither limb [36, 37]. One source of variation is that there is no standard for defining stronger vs. weaker limb in gait studies. Some researchers have made the distinction based on step length [33, 37] whereas others, like us, have used muscle strength [9, 34, 36, 44]. We chose to use LEMS to indicate which limb was weaker or stronger because the LEMS is a universally classified outcome measure that is understandable and testable by the majority of scientists and clinicians in the field. Based on this classification, our data showed that kinematic adaptations of the weaker limb were associated with improvements in skilled walking.

### Methodological considerations

In this analysis of secondary data collected as part of a pilot clinical trial, we were limited by the small data set available to us, which limits the generalizability of our results. Nevertheless, our results provide some insight into possible links between functional walking ability and inter-joint coordination that warrant further investigation. Another limitation is that kinematic parameters were derived during treadmill walking with BWS while the clinical walking measures of skill and speed were conducted over-ground with no BWS. However, the kinematics of treadmill walking and over ground walking are very similar [49, 50], suggesting that similar strategies are used during treadmill and level over ground walking. A recent study also showed that gait kinematics in individuals with iSCI are not altered by BWS [51]. Lastly, treadmill walking enables many more steps to be taken than over ground walking, which was critical for the kinematic analysis we conducted.

## Conclusions

Following gait training in individuals with m-iSCI, we observed that larger changes in hip-ankle inter-joint coordination and a decrease in knee range of motion during treadmill walking were associated with improvements in skilled walking. The changes in inter-joint coordination reflected some re-establishment of normative patterns as well as compensatory strategies, that varied across participants. No relationships were observed between the changes in joint range of motion or inter-joint coordination and walking speed. This study highlights the distinction of inter-joint coordination and range of motion on skilled walking as opposed to uninterrupted overground walking. Future studies should incorporate measures of inter-joint coordination, along with standard kinematic measures, during different walking tasks to further delineate the regulation of lower limb motor control associated with functional recovery after spinal cord injury.

## Data Availability

Contact correspondence author for data requests.

## Abbreviations

10MWT: 10-m walk test
AB: Able-bodied
BWS: Body-weight support
HA: Hip-ankle
HK: Hip-knee
KA: Knee-ankle
m-iSCI: Motor-incomplete spinal cord injury
ROM: Range of motion
ROM_A_: Ankle range of motion
ROM_H_: Hip range of motion
ROM_k_: Knee range of motion
SCI: Spinal cord injury
SCI-FAP: Spinal Cord Injury Functional Ambulation Profile
SSD: Square root of sum of squared differences
SSDHA_pre-post_: Square root of sum of squared differences between hip-ankle cyclograms pre- and post-training
SSDHK_pre-post_: Square root of sum of squared differences between hip-knee cyclograms pre- and post-training
SSDKA_pre-post_: Square root of sum of squared differences between knee-ankle cyclograms pre- and post-training
SSDnorm_post_: Square root of sum of squared differences between a SCI participants post-training cyclogram and the average able-bodied cyclogram
SSDnorm_pre_: Square root of sum of squared differences between a SCI participants pre-training cyclogram and the average able-bodied cyclogram
SSD_pre-post_: Square root of sum of squared differences between pre- and post-training cyclograms
